# Photobiomodulation invigorating collagen deposition, proliferating cell nuclear antigen and Ki67 expression during dermal wound repair in mice

**DOI:** 10.1007/s10103-020-03202-z

**Published:** 2020-11-27

**Authors:** Vijendra Prabhu, Bola Sadashiva Satish Rao, Anuradha Calicut Kini Rao, Keerthana Prasad, Krishna Kishore Mahato

**Affiliations:** 1 Department of Biophysics, Manipal School of Life Sciences, Manipal Academy of Higher Education, Manipal, Karnataka 576104 India; 2 Present Address: Department of Biotechnology, Manipal Institute of Technology, Manipal Academy of Higher Education, Manipal, Karnataka 576104 India; 3 Department of Radiation Biology and Toxicology, Manipal School of Life Sciences, Manipal Academy of Higher Education, Manipal, Karnataka 576104 India; 4 Present Address: Directorate of Research, Manipal Academy of Higher Education, Karnataka 576104 Manipal, India; 5 Department of Pathology, Kasturba Medical College, Manipal Academy of Higher Education, Manipal, Karnataka 576104 India; 6 Present Address: Department of Pathology, Yenepoya Medical College, Yenepoya (a Deemed to be University), Mangalore, Karnataka 575018 India; 7 Manipal School of Information Sciences, Manipal Academy of Higher Education, Manipal, Karnataka 576104 India

**Keywords:** Excisional wounds, Wound repair, Photobiomodulation, Proliferating cell nuclear antigen, Ki67, Collagen deposition

## Abstract

The present investigation focuses on understanding the role of photobiomodulation in enhancing tissue proliferation. Circular excision wounds of diameter 1.5 cm were created on Swiss albino mice and treated immediately with 2 J/cm^2^ and 10 J/cm^2^ single exposures of the Helium-Neon laser along with sham-irradiated controls. During different days of healing progression (day 5, day 10, and day 15), the tissue samples upon euthanization of the animals were taken for assessing collagen deposition by Picrosirius red staining and cell proliferation (day 10) by proliferating cell nuclear antigen (PCNA) and Ki67. The positive influence of red light on collagen synthesis was found to be statistically significant on day 10 (*P* < 0.01) and day 15 (*P* < 0.05) post-wounding when compared to sham irradiation, as evident from the image analysis of collagen birefringence. Furthermore, a significant rise in PCNA (*P* < 0.01) and Ki67 (*P* < 0.05) expression was also recorded in animals exposed to 2 J/cm^2^ when compared to sham irradiation and (*P* < 0.01) compared to the 10 J/cm^2^ treated group as evidenced by the microscopy study. The findings of the current investigation have distinctly exhibited the assenting influence of red laser light on excisional wound healing in Swiss albino mice by augmenting cell proliferation and collagen deposition.

## Introduction

Wound repair is an outcome of a series of well-orchestrated multiplex events to re-establish the skin’s anatomical and functional integrity. The process is grouped into overlapping phases of hemostasis, inflammation, proliferation, and remodeling. The repair process mainly becomes recurrently deficient due to extended trauma, prolonged infections, and inflammation [1]. In this line, extensive research is underway, focused on establishing better therapeutic modalities to expedite healing and improve patients’ quality of life.

Fibroblast migration and proliferation, synthesis of extracellular matrix (ECM), and successful granulation tissue formation are the hallmarks of the proliferative phase of the dermal repair process. Defects in the aforementioned events of the proliferative phase result in imperfective healing. Owing to its importance in the wound repair process, tracking markers of the proliferative phase becomes decisive to judge the therapy’s fate. Proliferating cell nuclear antigen (PCNA) and Ki67 are the well-established markers to probe cellular proliferation during wound repair. Ki67 is a nuclear protein associated with the cell cycle, synthesized by all proliferating cells of the active cell cycle and deficient in resting cells [2]. Ki67 antibodies specifically interact with intranuclear antigens of active proliferative phase cells. PCNA is another well-known nuclear protein, clinically trusted marker for proliferation, which participates in cell proliferation by mediating DNA polymerase. Elevated PCNA expressions were recorded during the S, G2, and M phases of the cell mitosis in healthy and cancerous tissues [3]. Blocking the PCNA production in cells severely affects cell division, indicating its importance in cell proliferation [4].

In the recent past, photobiomodulation (PBM) or low-power laser therapy (LPLT), a fully non-pharmacological pain-free approach, is earning popularity in the treatment of more than ninety ailments as investigated either in preclinical models or in clinical studies [5]. The indications inspected by PBM include a plethora of disorders of internal organs such as brain, bone, kidney, heart and lung, eye, connective tissue, muscle, and skin. In dermatology, PBM is administered mainly to treat delayed wound healing and hair regrowth and medications of psoriasis [5]. This treatment modality relies on low-intensity light of a wavelength in the visible to near-infrared (600–1070-nm) [6] from either coherent or quasi-coherent sources for best therapeutic effects [5, 7]. PBM’s positive outcomes at the cellular level have reported the involvement of numerous genes connected to mitochondrial signaling, cell attachment, cell movement, cell survival, cell proliferation, cell differentiation, and collagen synthesis [5, 8].

Currently, PBM is extensively being investigated in roughly 40 different countries [5]. Although most of the reports involving PBM have exhibited a positive effect on wound healing, a small number of inspections have proven the contrary, making its use uncertain in clinical practice [9, 10]. This disparity could be attributed to a lack of consensus on the fundamental mechanism of PBM and molecular targets of the therapy [11]. Moreover, PBM is a light-driven therapeutic modality; thus, choice of ideal illumination parameters including wavelength, irradiance, dose, time of light exposure, number of exposures, etc., are the predisposing factors that significantly control the outcome of the technique.

Recently, researchers reported an in-house PBM system for the optimization of fluence and time of irradiation to augment excisional skin repair in an animal model [12]. Uniform exposure of the irradiation source to the wound site was also demonstrated in that study [13]. Furthermore, histological evidence was also provided to elucidate the role of red light in regulating different phases of wound healing in Swiss albino mice [14]. Based on these reports, the present study is designed to interrogate the outcome of 632.8-nm laser illumination on the regulation of the proliferative phase (cell proliferation and collagen synthesis) during dermal wound healing in a preclinical model. Expressions of PCNA, Ki67, and collagen deposition were appraised following immediate and single exposure of different doses of the 632.8-nm laser.

## Materials and methods

### Selection of animals

For animal care and handling, instruction of the World Health Organization was followed. Institutional Animal Ethics Committee (IAEC/KMC/07/2007–2008) clearance was obtained before performing the experiments. Forty-five Swiss albino mice of both sexes (6–8 weeks old, weight 25–30 g) were used, which were housed in a regular pathogen-free condition with constant temperature (23 ± 2 °C), humidity (50 ± 5%), and light and dark cycle (14 and 10 h) respectively. During the study period, animals had free access to sterile food and water ad libitum.

### Full-thickness wound induction

Excisional wound induction upon anesthetization of animals was performed as per the previously described protocol [12]. Following wounding, the animals were housed in a separate polypropylene cage containing sterile paddy husk bedding.

### Laser irradiation of animal wounds

**Fig. 1:**
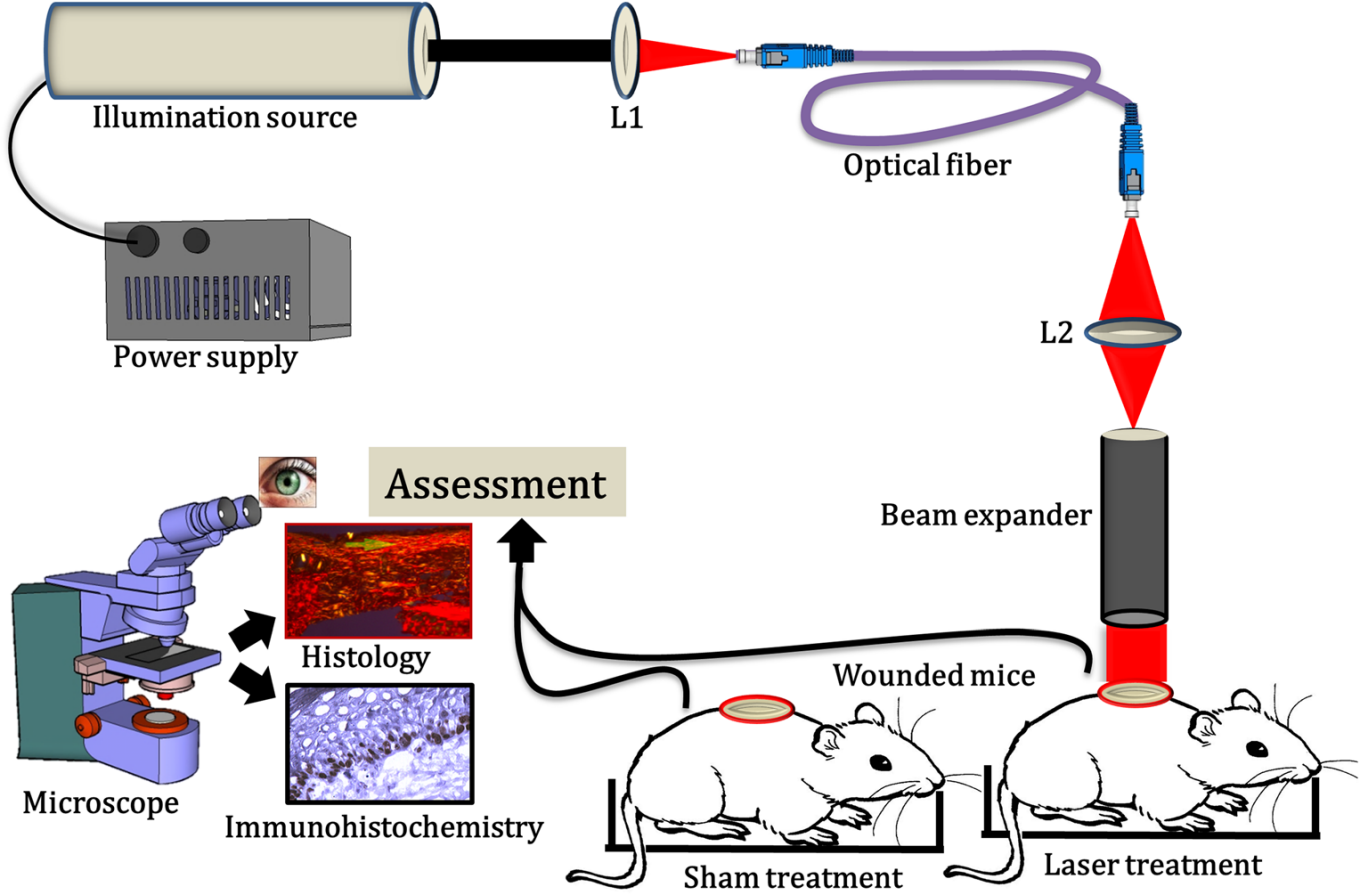
Schematic experimental setup used for treatment with PBM. The illumination source is He-Neon laser; L1 and L2 are the biconvex focusing lens

**Fig. 2:**
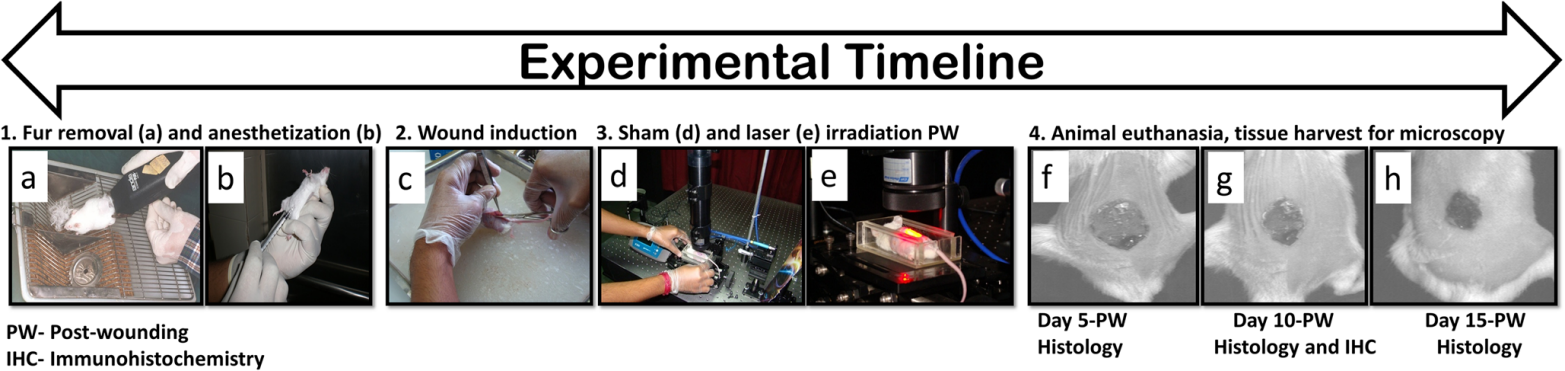
Experimental timeline indicating pre- and post-wounding procedures, treatment, and tissue sample collection intervals for microscopic analysis

Animals were illuminated after wounding to pre-assigned doses of the 632.8-nm laser. Figure 1 illustrates the graphical layout of the experimental setup, and Table 1 provides the details of the laboratory settings used for laser irradiation. The technical specifications of the experimental setup employed for the current investigation were described elsewhere [12]. Animals assigned to the sham irradiation (SI) group were not given laser or any other treatments. A brief description of the experimental timeline is illustrated in Fig. 2.

**Table 1:** Illumination parameters used for photobiomodulation study

Laser parameters	
Illumination source and wavelength (nm)	He-Ne laser; 632.8
The operating mode of the light source	Continuous wave (CW)
Type of polarization	Linear
Maximum output power (mW) of the laser	7
Power density (mWcm^−2^)	4.02
Fluences in J/cm^2^ (exposure time)	2 (8 min 31 s) 10 (42 min 40 s)
Number of laser exposure	Only once immediately following injury
Mode of laser exposure	Non-contact
The spot size of an expanded laser beam (cm)	1.5
Distance between beam expander and wound site (cm)	2
Room temperature (°C) during irradiation	23 ± 2
Room humidity (%) during irradiation	70

### Immunohistochemical (IHC) studies of wound granulation tissues

For IHC experiments, 15 animals were selected, further split into 3 groups of 5 animals. The first group was assigned to SI, the second group to 2 J/cm^2^ treatment, and the third group to 10 J/cm^2^ treatment. All the animals were euthanized on day 10 post-wounding to harvest wound granulation tissues. Excised tissues were immediately snap-frozen in liquid nitrogen.

### Microscopic inspection of granulation tissues

For microscopic examinations (histology), 30 animals were selected and split into 2 groups of 15 animals each. The first group was assigned to SI and the second group to 2 J/cm^2^ treatment. Five animals from each group were euthanized on the 5th, 10th, and 15th day post-wounding to harvest the granulation tissues and were immediately fixed in Bouin’s fixative for further tissue processing.

### Tissue processing for IHC studies

Around 3–5-μm thick sections were taken manually from the frozen granulation tissues. Endogenous peroxidase activity was blocked by treating the tissue sections with 3% hydrogen peroxide for 5–8 min at 4 °C. Subsequently, the sections were rinsed in phosphate-buffered saline (PBS-pH 7.4) for 5 min. Furthermore, the slides were covered with ready to use equilibrium buffer (iHistochem-Mouse Ki67 immunohistochemistry kit, cat. no. RUK-KI001) and incubated for 30 min in a moist chamber (for Ki67) or incubated with a ready to use blocking solution (Invitrogen PCNA Staining kit, cat. no. 93-1143) for 10 min at room temperature (for PCNA). The sections were successfully incubated overnight at 4 °C with the anti-Ki67 antibody (1:100 dilution with antibody diluent; iHistochem-Mouse Ki67 immunohistochemistry kit, cat. no. RUK-KI001) or with the anti-PCNA antibody (ready to use, Invitrogen PCNA Staining kit, cat. no. 93-1143) at 4 °C for 60 min. Later, the slides were rinsed with wash buffer and then with PBS, respectively. Furthermore, slides were incubated immediately either with streptavidin-peroxidase (Invitrogen PCNA Staining kit, cat. no. 93-1143) (for PCNA) for 10 min or ready to use Rabbit HRP polymer (iHistochem-Mouse Ki67 immunohistochemistry kit, cat. no. RUK-KI001) at 4 °C for 60 min (Ki67). For both Ki67 and PCNA detection, the slides were incubated with the substrate (diaminobenzedine tetrahydrochloride), followed by hematoxylin counterstaining (both substrate and hematoxylin are part of the kit). For negative control, tissue sections were incubated with PBS by omitting the primary antibody for Ki67 and PCNA. Colon cancer tissue sections (control slides—part of the kit) were used as a positive control. Cells with positive or no expression were stained either with brown and blue color, regardless of the color intensity. The stained slides were imaged using a digital camera fitted to an optical microscope (Motic BA 400, Motic Microsystems). A trained pathologist in a blinded fashion counted positive nuclei and total nuclei (both Ki67 and PCNA) in five random fields per section using image analysis software (Motic Images Plus 3.0, multifunctional microscopy software), and the mean for the same was computed. While counting the cells, hair follicles were excluded. For all the cell counting, a × 40 magnification objective lens was used. Quantification of PCNA and Ki67 levels was expressed as a percentage of positive cells in the tissue sections.

### Tissue processing for Picrosirius red (PSR) staining

PSR staining was performed on 5–7-μm thick tissue sections as per the previously published procedure [15]. For each slide, nuclear staining was performed by Weigert’s hematoxylin, followed by 0.1% PSR staining. A standard optical microscope with polarizing filters was used to capture collagen birefringence from the PSR-stained tissue sections. PSR-stained sections on different post-wounding days were photographed at × 10 and × 40 magnification objective lenses, and images were further used for image analysis.

### Image analysis for PSR staining

“TissueQuant” software was utilized to extract information on collagen birefringence from PSR-stained tissue sections [16], and image analysis was performed as described previously [17]. This software provided information on scores for assigned color (red/green/yellow) and pixel area. For the quantitative analysis, the mean and standard error of the mean scores were computed. The scores of red, yellow color (collagen type I), and green color (collagen type III) were added, and the sum of the scores was represented as percentage scores indicating the total collagen deposition. Total collagen deposited on day 5, day 10, and day 15 post-wounding for the SI and 2 J/cm^2^ treatment group was compared and plotted. Furthermore, to understand the possible role of red light in stimulating the early conversion of type III to type I collagen during the repair process, the individual contribution of type III and type I collagen was computed for SI and 2 J/cm^2^ groups on all post-wounding days. Total deposited collagen on each day for the respective group was considered as 100%. The contribution of either type I or type III collagen in total deposited collagen for each day for the respective group (SI and 2 J/cm^2^) was calculated and plotted. Type III and type I collagen contribution among SI and 2 J/cm^2^ were compared.

### Statistical analysis

Experimental data were represented as mean ± SEM. PCNA and Ki67 data of laser treatment and SI groups were compared for statistical significance by one-way analysis of variance with Bonferroni’s post hoc test utilizing GraphPAD Prism 4 software. Comparisons of total collagen values and individual contribution of collagen type I and type III among the SI and 2 J/cm^2^ groups were executed by Student’s unpaired two-tailed *t* test. Statistical significance was considered at *P* < 0.05.

## Results

### Immunohistochemical studies of wound granulation tissues

**Fig. 3:**
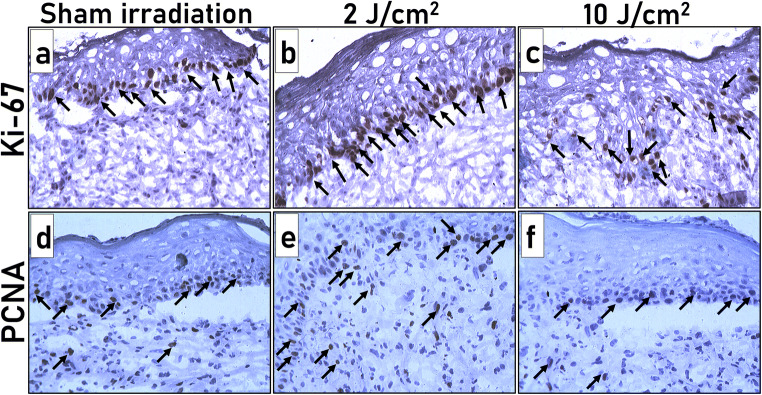
Ki-67 (a, b, and c) and PCNA expression (d, e, and f) in tissue sections of the laser treatment and SI group on day 10 post-wounding. **a**, **d** SI; **b**, **e** 2 J/cm^2^ treatment; **c**, **f** 10 J/cm^2^. Arrows indicate Ki67 and PCNA-positive cells displaying dark brown cells demonstrating the nuclear localization pattern (X 200)

Expression of Ki67 and PCNA from the granulation tissues of all the experimental groups on day 10 post-wounding was performed, and the results are presented in Fig. 3. A smaller number of Ki67-positive cells (brown cells; Fig. 3a) was recorded in the newly established epidermal layers in the SI group. Interestingly, immediate exposure of 2 J/cm^2^ after wounding a substantially elevated number of Ki67-positive cells in the freshly deposited basal layer of the epidermis was observed (Fig. 3b). On the contrary, a single bout of 10 J/cm^2^ showed fewer Ki67-positive cells (Fig. 3c). Equally, increased PCNA expression was noticed in 2 J/cm^2^ treated animals (Fig. 3e). Both SI (Fig. 3d) and laser treatment with 10 J/cm^2^ (Fig. 3f) were quite alike, showing a few PCNA-positive cells.

The SI group recorded 12.8 ± 1.49% positive cells, and 2 J/cm^2^ treatment groups showed 20.6 ± 2.13% positive cells for Ki67 (Table 2). Excision wounds subjected to 10 J/cm^2^ dose recorded with 11.2 ± 1.24% Ki67-positive cells. This elevated expression of Ki67 in 2 J/cm^2^ treated animals on the 10th day post-wounding was found to be statistically significant when matched to a SI group (*P* < 0.05) and to a single illumination of 10 J/cm^2^ treatment group (*P* < 0.01). In like manner, illumination of excision wounds with 2 J/cm^2^ exalted PCNA expression with 30.2 ± 2.00%. The percentage of PCNA-positive cells was lowered to 20.8 ± 1.59 and 19.8 ± 1.39 in SI and 10 J/cm^2^ treatment groups, respectively. This notable rise in expression of PCNA achieved by 632.8 nm at 2 J/cm^2^ was equally significant (*P* < 0.01) while compared to both SI and 10 J/cm^2^ treatment groups.

**Table 2:** Percentage of expression for Ki67 and PCNA in day 10 post-wound granulation tissues. Significance levels: for Ki67, *P < 0.05 compared to SI and **P < 0.01 compared to 10 J/cm^2^. For PCNA, **P < 0.01 compared to SI and 10 J/cm^2^

Sl. no	Proliferation marker	Percentage of expression		
		Sham irradiation (SI)	2 J/cm^2^	10 J/cm^2^
1	PCNA	20.8 ± 1.59	30.2 ± 2.00**	19.8 ± 1.39
2	Ki67	12.8 ± 1.49	20.6 ± 2.13*^,^ **	11.2 ± 1.24

### Microscopic inspection of granulation tissues

**Fig. 4:**
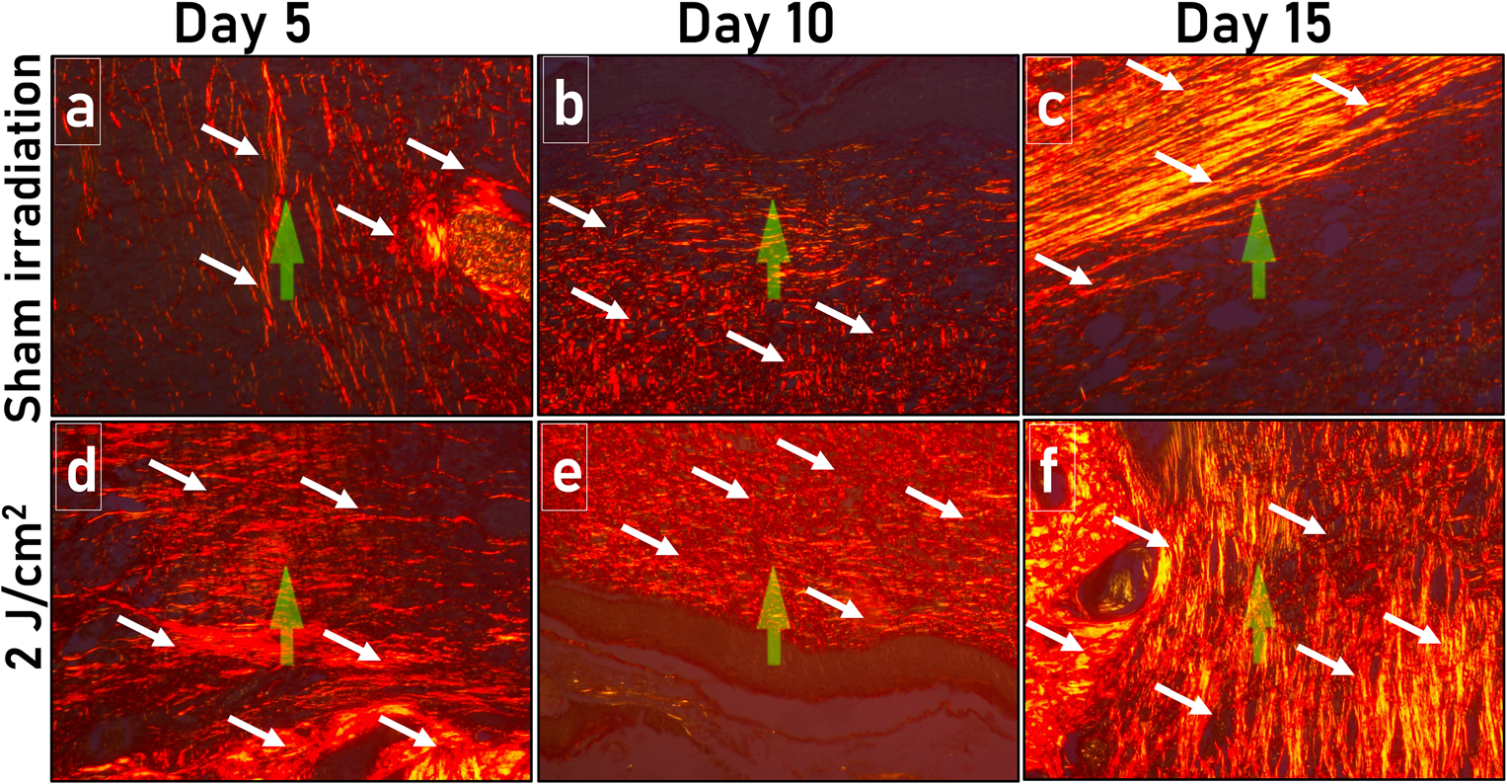
Photomicrographs of the SI (**a**, **b**, and **c**) and 2 J/cm^2^ treatment group (**d**, **e**, and **f**) on day 5, day 10, and day 15 post-wounding followed by PSR staining and visualization by the polarized microscope. Arrows indicate the collagen deposition in the histological sections (X 400)

**Fig. 5:**
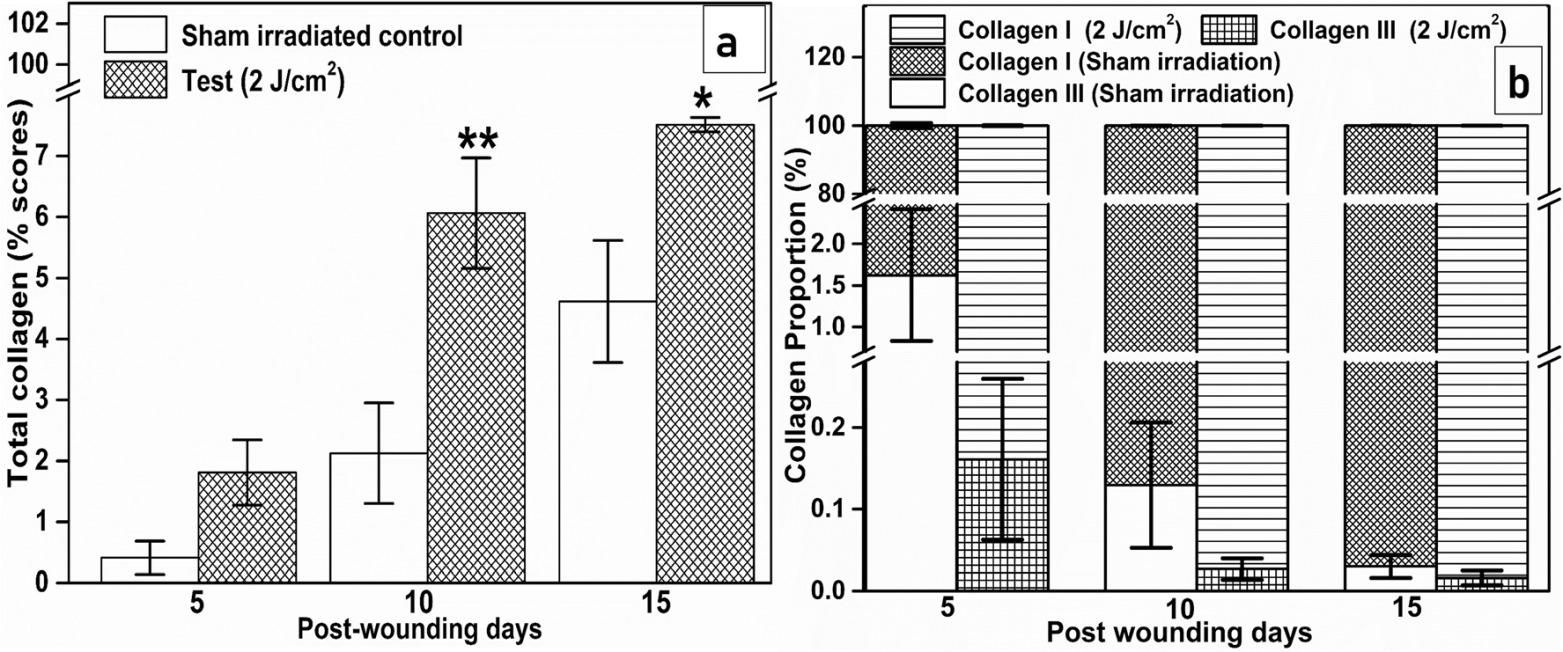
Comparison of collagen deposition among the SI and 2 J/cm^2^ treatment group. **a** Changes in the total collagen derived from image analysis of PSR-stained tissue sections. **b** Contribution of type III and type I collagen in SI and 2 J/cm^2^ treated animals on different post-wounding days. Data mean ± SEM. Level of significance *P < 0.05, **P < 0.01, and no symbol = insignificant compared to the SI group

The PSR-stained tissue sections of the SI (Fig. 4a, b, and c) and 2 J/cm^2^ treatment group (Fig. 4d, e, and f) on the 5th, 10th, and 15th day post-wounding were shown in Fig. 4. From image analysis of PSR-stained tissue sections, the total collagen (%) for SI and 2 J/cm^2^ treatment group on different post-wounding days was plotted (Fig. 5a). In the SI group, the total collagen values of 0.41 ± 0.27%, 2.12 ± 0.82%, and 4.61 ± 1.00% were noted on the 5th, 10th, and 15th day post-wounding, respectively. The 2 J/cm^2^ treatment noticeably raised the total deposited collagen with scores of 1.80 ± 0.53%, 6.06 ± 0.90%, and 7.51 ± 0.11% on the 5th, 10th, and 15th day post-wounding. This laser-assisted boost in total collagen was found to be statistically significant on day 10 (*P* < 0.01) and day 15 (*P* < 0.05) post-wounding when matched to SI. However, on day 5 post-wounding, the difference between SI and 2 J/cm^2^ treated animals was insignificant.

The percentage contribution of collagen III and collagen I in total deposited collagen for both the experimental groups on all the post-wounding time intervals was computed (Fig. 5b). On day 5 post-wounding, type III and type I collagen contribution for the SI was found to be 1.62 ± 0.79% and 98.39 ± 0.78%, respectively. For the same day, a single bout of 632.8-nm laser light at 2 J/cm^2^ dose reduced the type III collagen contribution to 0.16 ± 0.09% (*P* = 0.1046) and increased the type I collagen to 99.83 ± 0.098% (*P* = 0.1042). A similar trend was noticed for SI in type III collagen contribution with 0.12 ± 0.07% and 0.03 ± 0.01% compared to 2 J/cm^2^ treatment with 0.02 ± 0.01% (*P* = 0.2243) and 0.01 ± 0.01% (*P* = 0.4232) on day 10 and day 15 post-wounding respectively. Likewise, a higher contribution of type I collagen was recorded for the 2 J/cm^2^ treatment group with 99.97 ± 0.01% (*P* = 0.2240) and 99.98 ± 0.01% (*P* = 0.4233) compared to the SI group with 99.87 ± 0.07% and 99.97 ± 0.01% on day 10 and day 15 post-wounding, respectively. Although higher type III collagen contribution on day 5 post-wounding was recorded in the SI group when compared to the 2 J/cm^2^ treatment group, and higher type I collagen contribution was recorded in the 2 J/cm^2^ treatment group compared to SI in all post-wounding time intervals, these differences were not found to be statistically significant (*P* > 0.05).

## Discussion

In the past two decades, PBM has shown enormous progress in rejuvenating healing by conserving either the damaged or decaying tissue or cells under stress. PBM, a simple non-pharmacological approach with no reported cytotoxic effects, triggers various cellular and biological pathways without comprehensively accepted precise mechanisms [18]. Out of 3500 published research articles on PBM to date, 85%–90% of studies employed lasers as a light source [5]. Helium-Neon with the wavelength of 632.8-nm, gallium-aluminum arsenide, gallium arsenide, and aluminum gallium indium phosphide with a wavelength of 780, 820, 830, and 904-nm, respectively, are the most prominent lasers utilized for PBM studies [19]. Long visible and near-infrared light displayed decisive during tested wavelengths, while short wavelengths reported having adverse outcomes [20]. Lesser tissue scattering at a higher wavelength and least absorption by tissue components result in deeper tissue penetration at this spectral region (red to near-infrared). The present study was intended to inspect the possible photobiological mechanism to regulate proliferation stimulated by red light in a preclinical excisional wound model.

Ki67 is a non-histone nuclear protein associated with cell proliferation, even though its exact role in it was unknown yet. Ki67 antibodies are expedient in evaluating the proliferating cells in various normal, neoplastic, and regenerative tissues. Immunohistochemical identification of this protein for various pathological applications is very well accepted. The identification of Ki67 through IHC is considered a quick, reliable method to measure the cell population’s growth fraction under study [21]. Another non-histone nuclear protein PCNA, which is primarily expressed in the cycling cells, functions as the auxiliary protein for DNA polymerase δ essential for DNA synthesis in the S phase, and it intermingles with various cell cycle regulation and checkpoint proteins. The expression of these two markers was also examined concerning wound healing in preclinical models [22, 23].

In the current investigation, these two vital proliferation markers were selected to comprehend the role of red light irradiation in regulating proliferation in the course of the wound repair process. IHC findings indicated that an immediate illumination of 2 J/cm^2^ resulted in a 1.61% and 1.84% fold increase in Ki67-positive cells compared to SI and 10 J/cm^2^ laser dose, respectively. Likewise, a 1.45% and 1.53% fold increase in PCNA expression was recorded in 2 J/cm^2^ treatment compared to SI and 10 J/cm^2^ groups. A previous study by Wu and co-workers reported elevated PCNA expression leading to cellular proliferation during early tendon healing [24]. This study also suggested that PCNA could be an ideal marker to assess cellular proliferation during healing progression. Out of the two proliferative markers, PCNA-positive cells were higher than Ki67 cells in all experimental groups. This could be attributed to the expression of PCNA in resting cells and the longer half-life of PCNA, allowing it to recognize even after completion of cell division.

Recently enhanced proliferation in tenocytes was reported following low-level laser irradiation as measured by a substantial upsurge of Ki67 and PCNA expression [4]. Likewise, diode laser irradiation to periapical lesions in rats resulted in elevated expression of PCNA, leading to higher proliferation [25]. Similar studies performed by Wan-Ping and co-workers [26] reported increased cell proliferation via Ki67 expression in melanoma cells following single exposure of He-Ne laser. In a study reported by Gupta and co-workers [23] on a preclinical model of partial-thickness dermal abrasion, elevated PCNA expression has resulted in higher proliferation following 635-nm and 810-nm laser treatments. A study conducted by Li et al. on the rabbit skin wound model reported higher Ki67 expression resulting in rapid healing following red light-emitting diode treatment [27] compared to blue light and unilluminated control group. In the same way, high-frequency pulsed low-level diode laser therapy (904–910-nm) resulted in significantly higher PCNA expression in the tooth extracted preclinical wound model [28]. In an exciting finding reported by Shu and co-workers, scar fibroblast cells exposed to He-Ne laser at higher power densities (100 and 150 mWcm^−2^) lead to a rise in the number of cells in GO/G1 phase and reduction in cells in S phase measured by cell cycle assay [29]. In that study, high power He-Ne laser irradiation also impeded fibroblast proliferation through decreased PCNA expression in a preclinical hypertrophic scar model. The authors of the study directly linked the reduction of PCNA at higher power densities to the halting of cells in the G0/G1 phase. It was also concluded that optimal illumination parameters are critical in regulating the proliferation phase of tissue repair.

Outcomes of the current investigation are entirely in agreement with the previously published reports indicating the positive influence of red light on proliferation when applied at optimal doses [29, 30]. For the first time, the regulation of  Ki67 and PCNA following 632.8-nm laser exposure in a full-thickness excision wound model was reported. Furthermore, this study endorses the red light mediated proliferation during wound healing via Ki67 and PCNA. Thus, the current report’s outcomes postulate that 632.8-nm might promote the cells to traverse the G1 phase to enter the S phase and move through the G2/M checkpoint, thereby promoting cell proliferation during tissue repair. It might otherwise be indicated that an optimal dose of 632.8-nm laser promotes nuclear proteins’ expression to alter cell proliferation during wound repair.

Collagen, a vital constituent of the ECM, substantially contributes to the skin’s elastic property [31]. During wound repair, this vital protein mainly regulates cell proliferation and migration, and its role is supposed to be decisive in wound closure and successful wound healing [31]. The amount, quality, and collagen synthesis rate during repair are directly linked to the functional and esthetic outcomes of the newly formed tissue. Type I collagen is the most abundant ECM found in the skin, providing scar tissue resistance against friction [32]. Enhanced wound healing following PBM through elevated collagen synthesis has been reported in the literature [12–14]. Owing importance of collagen in wound healing, in the current investigation, it is proposed to scrutinize 632.8-nm laser’s role in the production of type I and type III collagen and total collagen deposition during the wound repair process.

The widely reported staining methods for collagen quantifications such as van Gieson and trichome fail to discern among different collagen types and identify thin fibers of collagen, resulting in underestimating collagen content [33]. Classical hematoxylin and eosin staining also extensively utilized for collagen quantification, although stains used have no specificity for this protein [34]. Quantification of collagen in PSR-stained tissue sections through bright-field microscopy was also reported previously [35]. However, this approach lacks to provide structural information and fails to differentiate between the thin and thick fibers (both appear red) and brightness, and fiber color is non-uniform. Thus, in the current investigation, the combination of PSR and polarized microscopy followed by image analysis for quantification of the contribution of type III and type I collagen and total deposited collagen post-laser treatment was performed. The wounded animals illuminated with 2 J/cm^2^ of red laser light displayed 4.40, 2.85, and 1.62fold increase in total collagen on day 5, day 10, and day 15 post-wounding compared to the SI group as revealed by the image analysis of PSR-stained tissue slices. Upon comparing the proportion of type I and type III collagen in total deposited collagen between the experimental groups, 10.10, 4.83, and 1.86 fold increase in type III collagen was recorded in SI compared to 2 J/cm^2^ treatment group on day 5, day 10, and day 15 post-wounding respectively. On the contrary, laser treatment with a 2 J/cm^2^ dose elevated the type I collagen formation compared to SI animals in all the tested post-wounding days. Even though total collagen deposition in 2 J/cm^2^ was significantly higher on day 10 and day 15 post-wounding, type I and type III collagen proportion comparison between the 2 J/cm^2^ and SI, the difference was found to be statistically insignificant.

Histological findings on collagen deposition following PBM therapy recorded in the present study thoroughly corroborated with previous investigations on biochemical measurements performed on granulation tissues at different post-wounding days [12]. In that study, biochemical estimation of hydroxyproline (a direct measure of collagen) in granulation tissues following single illumination of various laser doses of 632.8-nm at three different post-wounding treatment intervals was performed. The findings of the biochemical study concluded that red light illumination at 2 J/cm^2^ is the optimum dose for collagen deposition. Thus, only an optimal laser dose of 632.8-nm was selected for further microscopic investigations on collagen deposition in the present study.

Positive effects of red light (660-nm) on collagen deposition during the rats’ muscle repair process were reported previously [36, 37]. Similarly, the therapeutic effects of LEDs on collagen synthesis in a rodent model of skin wound healing are investigated by De Sousa et al. [38], wherein elevated fibroblast proliferation was reported. Kerppers and co-workers [39] irradiated incisional wounds on a rat model with 627-nm and 945-nm LEDs and reported prevalence of mature collagen in 627-nm treated wounds. In the same study, sizable raise in type I collagen post-infrared LED (945-nm) exposure was noticed compared to other treatments. Similarly, Pugliese and co-workers [40] inspected the impact of 670-nm laser on collagen and elastin fibers’ alterations during wound repair in a rat model. Wounds illuminated with 4 J/cm^2^ improved the deposition of collagen and elastin fibers on day 5 and day 7 post-wounding compared to non-irradiated control and 8 J/cm^2^ treatment group as endorsed by PSR staining of tissue sections. In that study, although authors noticed an improvement in the accumulation of these two ECM proteins following exposure to optimal laser dose during wound repair, the difference was statistically insignificant.

Equally, in the current exploration, experiential higher collagen deposition on day 5 post-wounding following red light illumination at 2 J/cm^2^ was not statistically significant compared to SI. While comparing the contribution, it was noticed that the SI group displayed higher type III collagen and lower type I collagen when matched to the 2 J/cm^2^ treatment group in all assessed treatment intervals. From the earlier pieces of literature, it is evident that type III collagen is predominant in early healing tissue, and at a later stage of remodeling collagen type I, overtake the former to expedite wound repair by avoiding the scar tissue formation [7, 32, 41]. The early replacement of type III collagen with type I collagen in healing tissue witnessed in the current inedited report could be linked to the exposure of an optimum dose of 632.8-nm laser, thereby augmenting the wound repair process. The findings of the current work on the early replacement of type III to type I collagen is entirely corroborated with an earlier published report [41]. Our assumption that a raise in collagen noticed in the current investigation is directly linked with an increase in the number of fibroblasts, as depicted in several other studies with other illuminated wavelengths [38, 42–45].

## Conclusion

In summary, the outcomes of the present study indicate that the 632.8-nm laser at optimum dose regulates the proliferative phase of wound repair in Swiss albino mice by altering the expression of proliferation markers (Ki67 and PCNA) and extending the exposure time did not seem to have a substantial influence on cellular proliferation. Our findings also established that PBM utilization at optimum dose-escalated total collagen production and prompted the early replacement of collagen III by collagen I in regenerated tissue, thus contributing to the hastened tissue repair process.
